# Evaluation of the effectiveness and safety of acupuncture in the treatment of premature ventricular contractions: A protocol for systematic review and meta-analysis

**DOI:** 10.1097/MD.0000000000027697

**Published:** 2021-11-05

**Authors:** Hongyu Li, Aidong Liu, Zhenhua Liu, Guangyu Cheng, Junfeng Cui, Shuangdi Li, Pengfei Li, Yuning Xin, Yinghui Liu

**Affiliations:** a Changchun University of Chinese Medicine, 1035 Boshuo Road, Changchun, Jilin, China; b The Third Affiliated Hospital of Changchun University of Chinese Medicine, 1643 Jingyue Street, Changchun, Jilin, China; c Affiliated Hospital of Changchun University of Chinese Medicine, 1478 Gongnong Road, Changchun, Jilin, China.

**Keywords:** acupuncture, meta-analysis, premature ventricular contractions, protocol, systematic review

## Abstract

**Background:**

Premature ventricular contractions are the most common type of arrhythmia. The clinical symptoms are mainly palpitations. In severe cases, syncope, angina pectoris and heart failure may occur, which seriously affect people's lives and ability to work. Antiarrhythmic drugs have many side effects and should not be taken for long periods. Acupuncture has a significant effect on the treatment of premature ventricular contractions. Therefore, to evaluate the effectiveness and safety of acupuncture in the treatment of premature ventricular contractions, we conducted this study, with the goal of providing a scientific methodology for this alternative treatment.

**Methods:**

We searched PubMed, Embase, Web of Science, Cochrane Library, China National Knowledge Infrastructure, Wanfang Database, China Science Journal Database, and China Biomedical Literature Database. We selected all randomized clinical trials related to the use of acupuncture in the treatment of premature ventricular contractions published on or before October 10, 2021, and we will conduct literature screening and data extraction based on specific inclusion and exclusion criteria. We will use the bias risk assessment tool from the Cochrane Systematic Review Manual to evaluate the quality of the research selected for inclusion in our study. RevMan5.3 software will be used to perform statistical analysis on the data.

**Results:**

The results of this study will provide evidence for the effectiveness and safety of acupuncture in the treatment of premature ventricular contractions.

**Conclusion:**

The purpose of this study is to explore the efficacy of acupuncture in the treatment of patients with premature ventricular contractions and to provide an effective reference for clinicians and patients on its use.

**INPLASY registration number:**

INPLASY2021100040.

## 1 Introduction

Premature ventricular contractions (PVCs) are heartbeats that occur below the bifurcation of the His bundle, prematurely depolarizing the myocardium.^[1]^ They are the most common arrhythmia. Among the general population, their prevalence on conventional electrocardiograms is estimated at 1% to 4%.^[2]^ PVCs are common in various organic heart diseases, such as coronary heart disease, rheumatic heart disease, cardiomyopathy, etc but also in nonorganic heart disease, such as autonomic dysfunction, emotional stress, electrolyte imbalance, etc.^[3]^ The clinical symptoms of PVCs have great variability, ranging from asymptomatic and mild heart palpitations to premature beats triggering malignant ventricular arrhythmia and causing syncope, or blackouts, which seriously affect people's lives and work.^[4]^

At present, the commonly used drugs for the treatment of PVCs in Western medicine include betablockers, mexiletine, and amiodarone, etc. These drugs can correct the original arrhythmia, but they can also induce new arrhythmias, thus affecting the treatment effect of patients, and should not be taken for long periods of time.^[5]^ In contrast, traditional Chinese medicine offers obvious advantages. Existing studies have shown that acupuncture, Chinese patent medicines, and Chinese herbal medicines have significant positive effects in treating PVCs, not only regulating the overall function of the patient's body but also reducing the side effects experienced with Western medicine.^[6–12]^ A systematic review of the treatment of PVCs with traditional Chinese medicine has been published, but there is no systematic review of the effectiveness of acupuncture in the treatment of PVCs. Therefore, we conducted this research to provide a scientific reference for the alternative treatment of PVCs using acupuncture.

## 2 Methods and analysis

### 2.1 Information sources and search strategy

This study was prepared in accordance with the guidelines of the preferred reporting items for systematic reviews and meta-analyses protocols. This protocol is meant for a systematic review and meta-analysis of previously published studies, so it does not require patient and public participation, nor does it require ethical approval. We will search foreign language literature databases such as PubMed, Embase, Web of Science, and the Cochrane Library as well as Chinese literature databases such as the China National Knowledge Infrastructure, Wanfang Database, China Science Journal Database, and China Biomedical Literature Database, using the keywords “acupuncture” and “premature ventricular contractions”. Our aim is to find randomized clinical trial (RCT) articles on the use of acupuncture in the treatment of PVCs. The search time frame was from the establishment of each of the databases through October 10, 2021. The prospective registration has been approved by the International Platform of Registered Systematic Review and Meta-analysis Protocols (https://inplasy.com/inplasy-2021-10-0040/) under registration number inplasy2021100040.

### 2.2 Inclusion criteria

The literature included in our study met the following criteria: the patients met the internationally recognized diagnostic criteria for PVCs, regardless of race, age and sex; the study type was an RCT; the experimental group received acupuncture treatment alone or acupuncture combined with treatment by conventional Western medicine; and the control group was a blank control group, a placebo group, or received treatment only through conventional Western medicine.

### 2.3 Exclusion criteria

Exclusion criteria were as follows: those studies with participants who did not meet the diagnostic criteria of PVCs or with participants with severe complications; non-RCTs research; studies with duplicate data or incomplete data; case reports, animal experiments, research protocols, reviews, and conference summaries, etc.

### 2.4 Outcome measures

The primary outcome indicators were clinical symptom curative effects, ECG curative effects, and total number of PVCs in 24 hour dynamic electrocardiograms. Secondary outcome indicators were Traditional Chinese Medicine syndrome curative effects, Traditional Chinese Medicine syndrome scores, heart rate improvement, and adverse reactions.

### 2.5 Literature screening and data extraction

The literature screening and data extraction will be performed independently by 2 researchers (HL and ZL). First, they will read the titles and abstracts of each article, conducting a preliminary screening to exclude duplicate articles and documents that are not related to the subject research; then they will read the full text of each article to determine whether the study can be included according to the inclusion and exclusion criteria; finally, they will extract information from the articles selected for inclusion in the current study. The extracted information will include the first author, publication year, number of cases, sex, age, intervention measures, treatment type, treatment course, outcome indicators, bias risk assessment, and research results. In the process of literature screening and data extraction, the 2 researchers will independently complete the data extraction, and then each will hand over their data to the other for inspection. If there are differences of opinion, they will be resolved through internal negotiation or discussion with the third researcher (YL).

### 2.6 Risk of bias in assessment

Two researchers (PL and YX) will independently apply the bias risk assessment tool provided by Cochrane System Reviewer Manual 5.1.0 to evaluate the quality of the included research literature. Specific content to be assessed includes random sequence generation, allocation hiding, blinding of patients, researchers and outcome evaluators, completeness of results data, selective reporting and other biases. These studies will be divided into 3 quality levels: high risk of bias, low risk of bias, and unclear risk of bias. If there is a disagreement, it will be resolved through internal negotiation or through discussion with a third researcher (YL).

### 2.7 Statistical analysis

We will use RevMan5.3 software (Cochrane Collaboration) to analyze the data. The effect value of the count data uses the relative risk, and the effect value of the measurement data uses the standardized mean difference, which is represented by a 95% confidence interval. We will use a *P* value and *I*^2^ to evaluate the heterogeneity in the results of the study: *I*^2^ < 50% and *P* > .05 indicates that the level of heterogeneity is low, and the fixed effects model will be used for analysis; *I*^2^ ≥ 50% and *P* ≤ .05 indicates existence significant heterogeneity, and the select random effects model will be used for analysis. Subgroup analysis or sensitivity analysis will be performed according to the possible causes of heterogeneity.

### 2.8 Subgroup analysis and sensitivity analysis

We will conduct a subgroup analysis of factors that may lead to sources of heterogeneity, such as the number of cases included in the literature, age, intervention measures, treatment types, and duration of treatment. If possible, we will conduct a sensitivity analysis to assess the robustness of the included results. If the results are unstable, studies with a high risk of bias will be excluded.

### 2.9 Reporting biases assessment

If more than 10 articles will be included in the study, we will use a funnel chart to assess reporting bias. At the same time, Egger and Begg tests will be performed using Stata 12.0 software (Stata Corp). If *P* > .05, then it will mean there is no publication bias.

### 2.10 Ethics and dissemination

The data included in this study will all be obtained from published articles, so ethical approval is not required. We will submit the final research results to a peer-reviewed journal for publication.

## 3 Discussion

PVCs are the most common arrhythmia. The clinical symptoms are mainly palpitations. In severe cases, syncope, angina pectoris, and heart failure may occur, which severely reduce the patient's quality of life and impact a patient's ability to work. Antiarrhythmic drugs have serious side effects and should not be taken for long periods of time. As a more convenient and safer treatment method, acupuncture can effectively relieve the symptoms of PVCs and reduce the occurrence of adverse events.

Acupuncture is an important part of traditional Chinese medicine; it has a long history and a wide range of applications. It has a low incidence of adverse reactions, it is a simple procedure, and its cost is low. It can dredge meridians, run qi and blood, reconcile yin and yang, enhance physical fitness, and it has significant clinical effects. However, there are still relatively few studies on the treatment of PVCs using acupuncture. Therefore, this study will systematically evaluate the effectiveness and safety of acupuncture treatment of PVCs and provide evidence-based medical guidance for the treatment of PVCs with acupuncture. Our study will enable clinicians and patients to have a deeper understanding of the efficacy of acupuncture in the treatment of PVCs and will provide new ideas and methods for the treatment of PVCs.

## Author contributions

**Conceptualization:** Hongyu Li, Zhenhua Liu

**Data curation:** Hongyu Li, Pengfei Li, Yuning Xin

**Formal analysis:** Hongyu Li, Pengfei Li, Yuning Xin

**Funding acquisition:** Aidong Liu, Yinghui Liu

**Investigation:** Guangyu Cheng, Shuangdi Li

**Methodology:** Junfeng Cui, Shuangdi Li

**Project administration:** Guangyu Cheng, Junfeng Cui

**Supervision:** Aidong Liu, Yinghui Liu

**Validation:** Hongyu Li, Zhenhua Liu

**Writing – original draft:** Hongyu Li

**Writing – review & editing:** Hongyu Li, Aidong Liu, Yinghui Liu
